# Cytocompatibility of titanium and poly(etheretherketone) surfaces after O_2_ non-thermal plasma sterilization

**DOI:** 10.1371/journal.pone.0290820

**Published:** 2023-08-30

**Authors:** Christina Maillet, Florence M. Klein, Florian Le Bras, Frederic Velard, Christine Guillaume, Sophie C. Gangloff, Marie-Paule Gelle

**Affiliations:** 1 BIOS EA 4691, Biomatériaux et Inflammation en Site Osseux, SFR CAP Santé, FED 4231, Université de Reims Champagne Ardenne, Reims, France; 2 UFR Odontologie, Université de Reims Champagne Ardenne, Reims, France; 3 Pôle de Médecine Bucco-Dentaire, Centre Hospitalier Universitaire de Reims, Reims, France; 4 UFR Pharmacie, Aurora Cold Plasma Sterilisation, Reims, France; 5 UFR Pharmacie, Université de Reims Champagne Ardenne, Reims, France; Université de Rouen Normandie, FRANCE

## Abstract

The sterilization of medical devices is paramount to achieve an acceptable level of sterility assurance and to prevent hospital-acquired infections. However, some medical devices cannot be sterilized by usual processes such as autoclave (AC) and gamma-ray irradiation (GI). A new non-thermal plasma (NTP) process using sealed bag that preserves the sterile state of the devices could be used as an alternative sterilization method. The aim of the study was to assess the cytocompatibility of titanium and poly(etheretherketone) (PEEK) surfaces after O_2_-NTP sterilization compared to GI and AC. MG-63 osteoblast-like cells were seeded on titanium (TA6V) and PEEK disks sterilized by AC, GI and O_2_-NTP. The cells’ viability and proliferation, determined by WST-1 and DNA quantification respectively, were enhanced whatever the material types from 3 to 10 days. When seeded on titanium, MG-63 cells showed a higher viability and proliferation after GI and O_2_-NTP treatment compared to AC treatment. When cultured on PEEK, MG-63 cells showed a higher viability after O_2_-NTP treatment. No difference of proliferation was observed whatever the sterilization processes. The cell colonization of the materials’ surface was confirmed by scanning electron microscopy. Lactate dehydrogenase (LDH) assay revealed no cytotoxicity. Thus, O_2_-NTP led to similar cell responses to AC and GI and could be a cost-effective alternative process to the usual sterilization methods for fragile medical devices.

## Introduction

Titanium and poly(etheretherketone) (PEEK) have been widely used as implant materials due to their biocompatibility. Although titanium presents an excellent corrosion resistance on its oxide surface, it might provoke allergic reactions and shift color due to exposure. PEEK, a semi-crystalline polyaromatic linear thermoplastic polymer, is an alternative to metallic implants such as spinal cord stimulation devices. This material presents many advantages such as a low allergenic potential, an elastic modulus comparable with cortical bone [1], and an absence of corrosion [2–5].

Before implanting in the body, these materials must be sterilized according to the ISO 14937 norm [6]. Sterilization is a physical or chemical method used to eliminate or kill microorganisms including bacteria, spores, fungi, and viruses. The optimum procedure should sterilize the material without altering its functionality, its surface topography or its biomechanical and biocompatible properties [3, 4, 7–11]. Many techniques of sterilization exist such as autoclave (AC), gamma-ray irradiation (GI), and ethylene oxide gas. The autoclave, a physical method based on saturated vapor, is the most recommended method of sterilization [12] that destroys the microorganisms’ metabolic and structural components [13]. However, it could not be recommended for heat-sensitive materials due to the high temperature of this process [14–16]. Gamma-ray irradiation sterilization process uses cobalt-60 irradiation to damage components of microorganisms on a wide variety of medical devices [17]. However, some materials can be degraded or crosslinks can be formed, altering their structure [12]. Ethylene oxide is a chemical process based on gas diffusion effective at low temperature with a high penetration in a wide range of materials. However, this technique needs a long desorption time to avoid any toxicity risk for the handler and the patient. As some carcinogenic risks for patients have been described, its use is no longer recommended [12, 13].

Consequently, non-thermal plasma (NTP) has been studied as an alternative standard sterilization process for many decades [3, 4, 18]. Plasma is an ionized gas, consisting of electrons, ions, ultraviolet photons, neutral species as well as reactive oxygen and nitrogen species. These elements could break covalent bonds and thus initiate various chemical reactions leading to the alteration of microorganisms. Many NTP processes are described in the literature such as dielectric barrier, plasma jet, corona discharge… Each technique differs from one another in terms of operating pressure (low or atmospheric pressure) and operating conditions (electric current, gas nature and flow rate) [3, 19–22]. But, if those processes lead to the inactivation of microorganisms (bacteria, spores, viruses, and fungi), they cannot preserve the sterile state and the safety of the treated device after the end of the processes. To overcome this issue, a low-pressure plasma sterilization process, where plasma was generated inside a sealed bag containing the devices, was developed enabling the preservation of the sterile state [23–25].

Thus, a large number of studies have described the efficiency of NTP on microorganisms inactivation. Other authors also studied the efficiency of this process in order to modify the surface of materials (titanium, zirconia, PEEK), and consequently to improve the adherence and proliferation of osteoblast or fibroblast cells [4, 9–11, 18, 26, 27]. However, few of these studies concerned the biocompatibility of an implant device treated by NTP [4, 9, 10, 27]. They showed that oxygen-NTP (O_2_-NTP) did not alter cell viability (osteoblasts or gingival fibroblasts of murine or human origin) and could even improve cell proliferation on different implant materials.

The aim of the present study was to evaluate on MG-63 the cytocompatibility of TA6V titanium and PEEK disks after sterilization in a sealed bag by O_2_-NTP treatment and to see if it had lower toxic effects compared to gamma-ray irradiation and autoclave sterilizations.

## Materials and methods

### Materials

TA6V titanium (grade 5) and PEEK disks were donated by the Centre Régional d’Innovation et de Transfert de Technologie (CRITT-MDTS, Charleville-Mézières, France). A total of 324 disks (12 mm in diameter and 4 mm thick) for each material were randomly divided into three groups (n = 108) according to three methods of sterilization: autoclave (AC), gamma-ray irradiation (GI), and low-pressure oxygen non-thermal plasma (O_2_-NTP).

### Disks sterilization

Cleaned disks were packed in sealed sterilization bags (Sterilsop^®^, Dutscher, France; SüdPack^®^ Medica, Germany) and sterilized by gamma-ray irradiation, autoclaving, or O_2_-NTP. The conditions of each sterilization method were as follows: AC at 135°C for 20 min (AVX 6131, SMI, Montpellier, France); GI performed at 25 kGy by Ionisos (Dagneux, France); and O_2_-NTP for 120 min [23]. The parameters of O_2_-NTP were: O_2_ flow rate at 1 sccm, radio-frequency powered at 100 W, and magnetic coils at 14 Gauss. During all the process, the temperature indicator showed that it was inferior to 40°C inside the sealed bags.

### Cell culture

Human osteoblast-like MG-63 cells (ATCC CRL-1427, Manassas, VA, USA) were cultured in Dulbecco’s modified eagle medium (DMEM) (Gibco^™^, ThermoFisher Scientific, Praisley, UK) supplemented with 10% (v/v) fetal bovine serum (Pan Biotech, Dutscher, France), and 1% penicillin/streptomycin (Gibco^™^, Fisher Scientific, France). Once at confluence, cells were collected, centrifuged, rinsed with PBS and resuspended in 5 mL of DMEM [28]. The cell concentration was adjusted to 10^5^ cells/mL and then, 100 μL were gently seeded on the center of the sterilized TA6V and PEEK disks in 24-well plates (Dutscher, Thermo Scientific Numc, Bernolsheim, France; Ø 18 mm) (10^4^ cells/disk). To promote cell adhesion, disks were incubated at 37°C in a humidified 5% CO_2_/95% air atmosphere incubator (ThermoFisher Scientific, France) for 3 hours. Then, 900 μL of DMEM were added in each well, before incubation for 3, 7 or 10 days. Cells seeded directly into the plastic well were used as negative control.

### Cell viability analysis

After each cultured period, the viability of MG-63 osteoblast cells was analyzed using a colorimetric assay to quantify the cleavage of the water soluble tetrazolium by mitochondrial dehydrogenases (WST-1, Roche Diagnostics GmbH, Mannheim, Germany) according to manufacturer’s instruction. The absorbance at OD 440 nm was measured using a multi-well spectrometer (FLUOstar Omega, BMG Labtech, France) and corrected at 750 nm to remove the background.

### Cell proliferation

The proliferation of MG-63 cells was determined after 3, 7 and 10 days of incubation. The extraction of DNA was assessed using Maxwell^®^ 16 LEV Blood DNA kit (Promega, France) (Plate-forme Régionale de Biologie Innovante (PRBI), Centre Hospitalier Universitaire, Reims, France) according to the manufacturer’s instructions. The quantification of DNA was performed with picodrop using an elution buffer as blank.

### Cytotoxicity assessment

Cytotoxicity was assessed by lactate dehydrogenase (LDH) assay after 3, 7 and 10 days of incubation. For each well, 50 μL of the culture supernatant was incubated with 50 μL of reaction mix for 28 minutes in the dark and at room temperature (cytotoxicity detection kit (LDH), Roche Diagnostics GmbH, Mannheim, Germany). The absorbance was measured using a multi-well spectrometer (FLUOstar Omega, BMG Labtech, France) equipped with 492 nm and 700 nm filters. As positive control, 20 μL of triton X-100 (Triton^®^ X-100, Acros Organic, Geel, Belgium) were added to cells in wells containing 1 mL of medium to reach a final concentration of 2% triton.

### Scanning electron microscopy (SEM)

SEM observations were set to visualize the proliferation of MG-63 cells on both material surfaces after 3 and 10 days of incubation. The disks were fixed with 2.5% glutaraldehyde in phosphate buffer solution (PBS) for 1h at room temperature, and rinsed two times with PBS for 10 min. The samples were dehydrated in graded series of ethanol/water solutions at 50%, 70%, 90% and 100% (twice) then covered with hexamethyldisilazane solution (Sigma-Aldrich, St. Louis, MO, USA) [23]. The samples were desiccated overnight at room temperature then placed at 37°C. The samples underwent a gold sputtering process (around 10 nm thick) using a JEOL JFC 1100 ion sputter. Cells were then observed with a SEM (JEOL, JSM-7900F, Croissy Sur Seine, France).

### Statistical analysis

Each assay was performed using duplicate wells in triplicate measurements. Each experiment was repeated six times independently. Data were analyzed with GraphPad Prism 8.0.2 (GraphPad Software, La Jolla, CA). The Shapiro-Wilk test revealed a non-normal distribution. Then, a pair-wise comparison was performed between the independent groups using the non-parametric Mann-Whitney test. For all results, significant differences were defined with p-values less than 0.05.

## Results

### Cell viability

**Fig 1:**
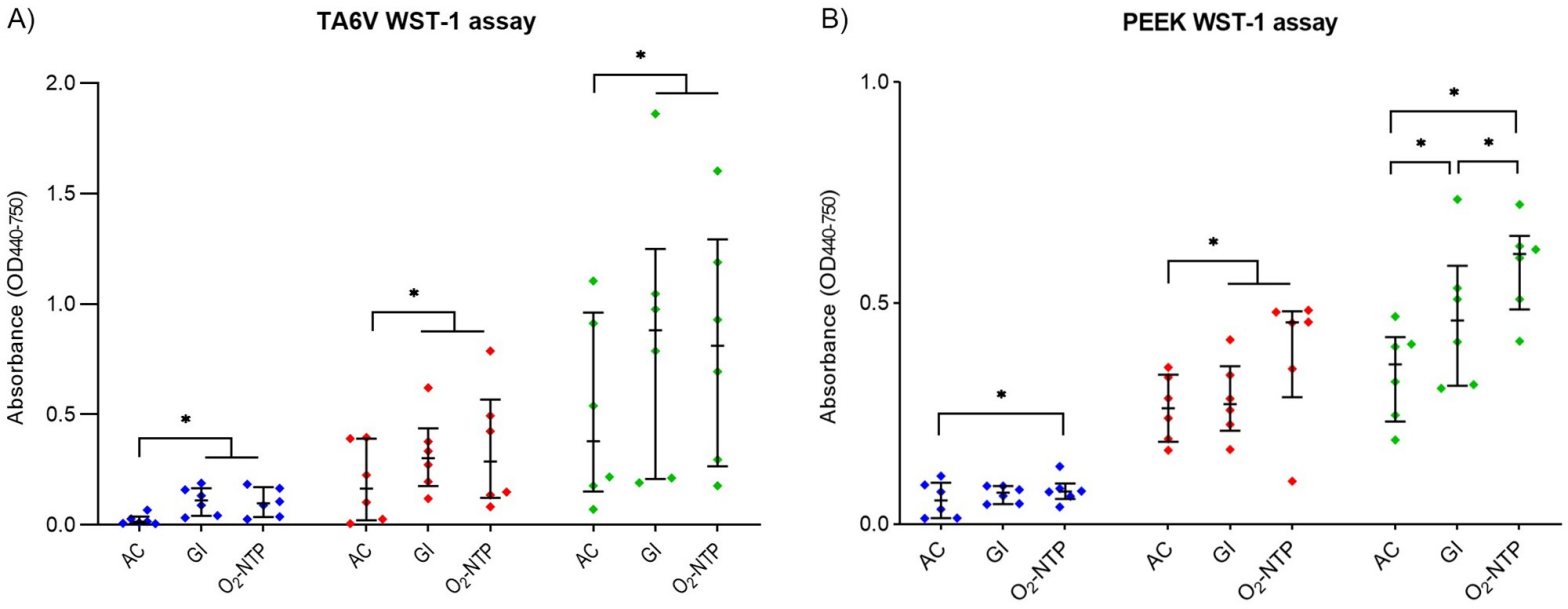
Viability of MG-63 cells seeded on sterilized disks of TA6V (A) and PEEK (B) after 3 (blue), 7 (red), and 10 (green), days of culture. Disks were sterilized by autoclave (AC), gamma-ray irradiation (GI), or oxygen non-thermal plasma (O_2_-NTP) (p-values of p<0.05 (*) were considered statistically significant).

Fig 1 shows the viability of MG-63 cells seeded on TA6V and PEEK disks sterilized by AC, GI or O_2_-NTP (Fig 1A and 1B). The cell viability increased from 3 to 10 days of culture whatever the sterilization method and the biomaterial (Fig 1A). Concerning the sterilization of TA6V, GI and O_2_-NTP treatment led to a significantly higher cell viability than AC (p<0.05) whatever the day of culture. In contrast, on sterilized PEEK disks and after 3 days of culture, the cell viability was statistically higher after O_2_-NTP treatment than AC process (p<0.05) (Fig 1B). At day 7, a significantly higher cell viability was noted after O_2_-NTP compared to AC and GI treatments (p<0.05). After 10 days of culture, the cell viability was significantly different between the three sterilization treatments. O_2_-NTP led to a significantly higher cell viability than GI (p<0.05), which was significantly higher than AC (p<0.05).

### Cell proliferation

**Fig 2:**
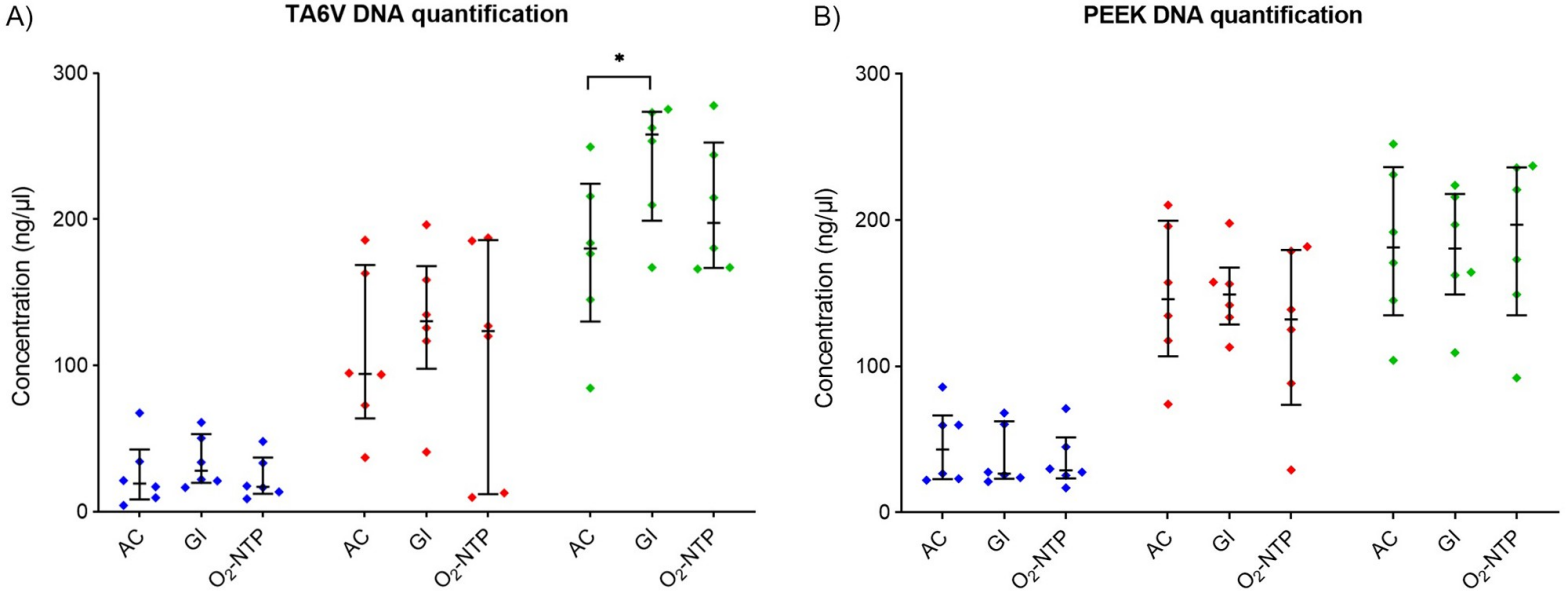
Proliferation of MG-63 cells seeded on sterilized disks of TA6V (A) and PEEK (B) after 3 (blue), 7 (red), and 10 (green), days of culture. Disks were sterilized by autoclave (AC), gamma-ray irradiation (GI), or oxygen non-thermal plasma (O_2_-NTP) (p-values of p<0.05 (*) were considered statistically significant).

The proliferation of MG-63 cells was assessed by quantifying DNA after seeding cells on TA6V and PEEK disks sterilized by AC, GI or O_2_-NTP (Fig 2). For both materials, the DNA quantity increased with time whatever the sterilization process (Fig 2A and 2B). Also, there was no significant difference of cell proliferation whatever the sterilization process at days 3 and 7 (Fig 2A and 2B). Concerning TA6V, only GI treatment led to a significantly higher cell number compared to AC (p<0.05) after 10 days of culture. There was no significant difference after GI and O_2_-NTP treatments (Fig 2A). Concerning the PEEK, after 10 days, there was no significant difference in cell proliferation whatever the sterilization methods (Fig 2B).

### Cytotoxicity

**Fig 3:**
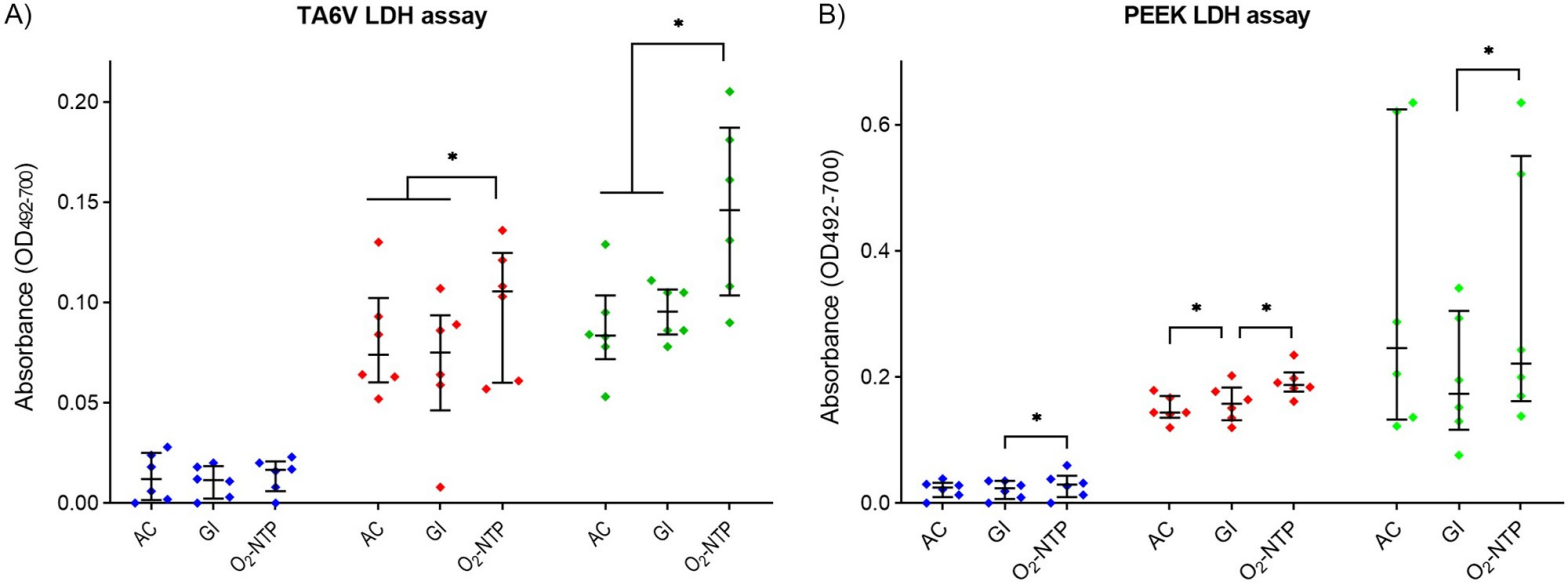
Cytotoxicity of sterilized disks of TA6V (A) and PEEK (B) on MG-63 cells after 3 (blue), 7 (red), and 10 (green), days of culture. Disks were sterilized by autoclave (AC), gamma-ray irradiation (GI), or oxygen non-thermal plasma (O_2_-NTP) (p-values of p<0.05 (*) were considered statistically significant).

Cytotoxicity was assessed by LDH assay after 3, 7 and 10 days of incubation of MG-63 cells seeded on sterilized disks of TA6V and PEEK (Fig 3). For both materials, the cytotoxicity increased from 3 to 10 days whatever the sterilization process (Fig 3A and 3B). Concerning TA6V, no significant difference was observed between the sterilization treatments after 3 days of culture (Fig 3A). At day 7 and 10, AC and GI led to a significantly lower cytotoxicity than O_2_-NTP (p<0.05). Concerning PEEK, a significant difference of cytotoxicity was observed between GI and O_2_-NTP treatments whatever the day of culture (p<0.05) (Fig 3B). Also, AC sterilization led to a significantly lower cytotoxicity than GI treatment after 7 days of incubation (p<0.05).

### SEM

**Fig 4:**
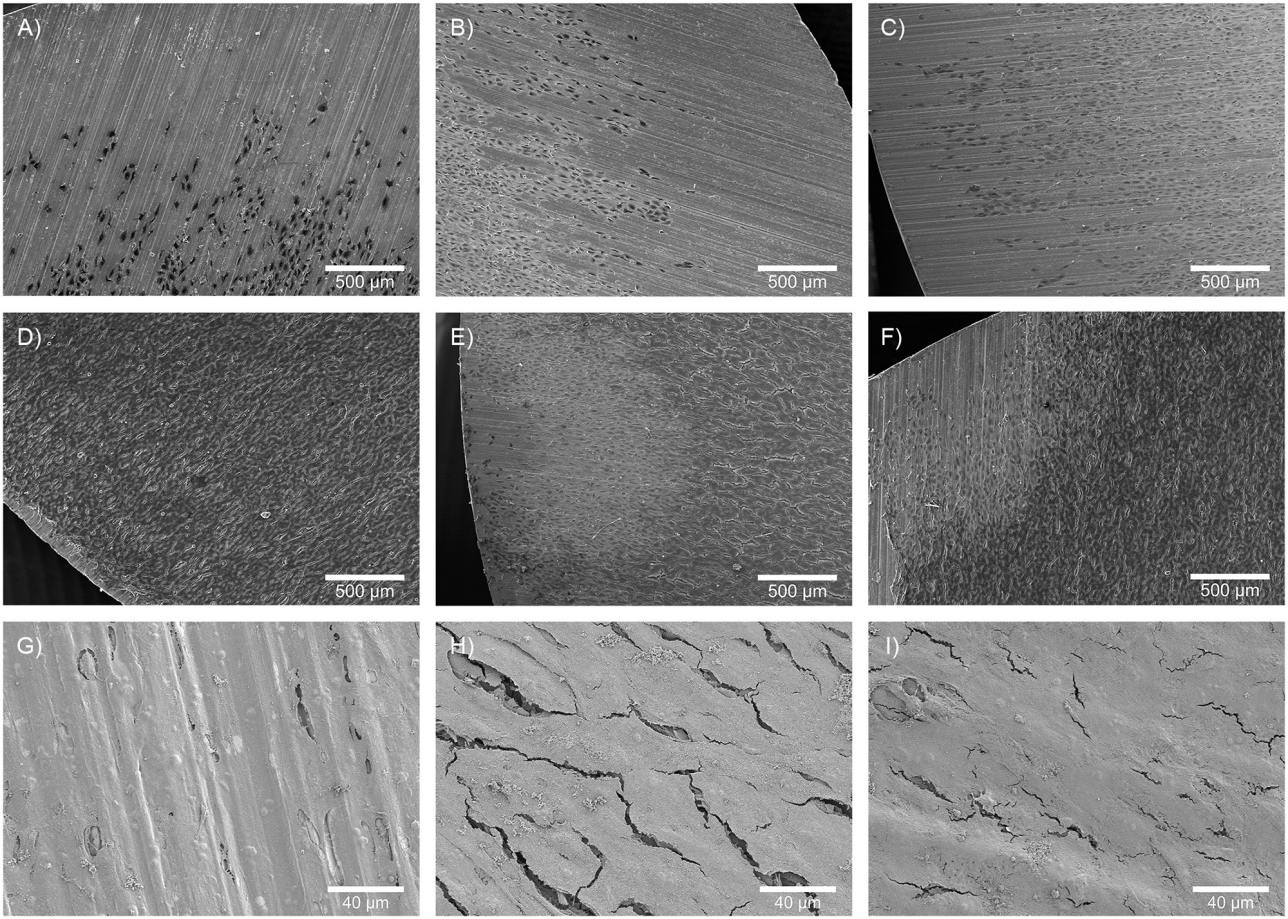
SEM images of MG-63 cell proliferation on disks of TA6V sterilized by different processes. On culture day 3, cells were located in the center of the disks sterilized by (A) autoclave (AC), (B) gamma-ray irradiation (GI), (C) or oxygen non-thermal plasma (O_2_-NTP). On culture day 10: cells spread over almost the entire surface of the disks sterilized by AC (D), GI (E) or O_2_-NTP (F); and less cellular multilayers after AC (G) were observed in comparison to GI (H) and O_2_-NTP (I).

**Fig 5:**
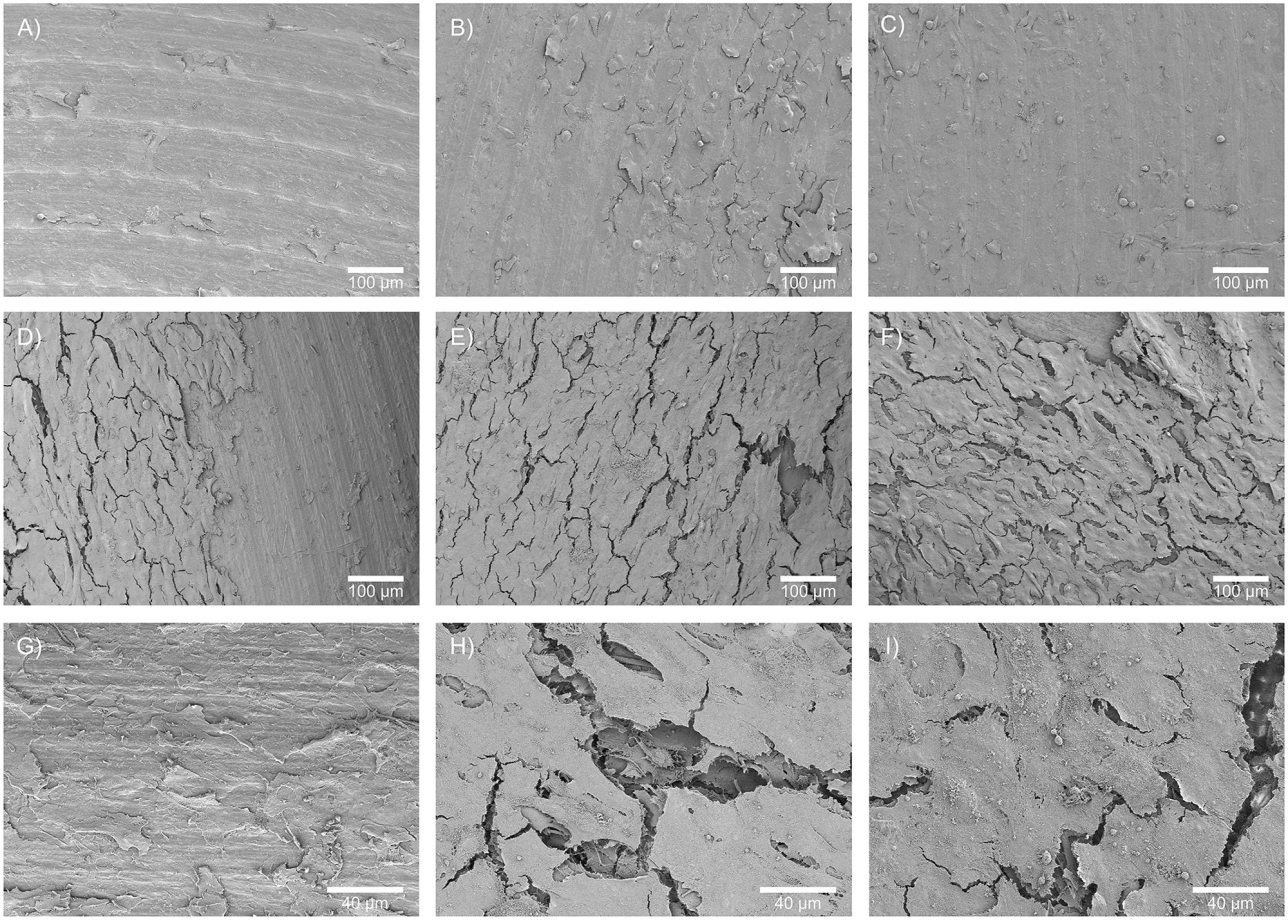
SEM images of MG-63 cell proliferation on disks of PEEK sterilized by different processes. On culture day 3, cells were located in the center of the disks sterilized by (A) autoclave (AC), (B) gamma-ray irradiation (GI), (C) or oxygen non-thermal plasma (O_2_-NTP). On culture day 10: cells spread over almost the entire surface of the disks sterilized by AC (D), GI (E) or O_2_-NTP (F); and less cellular multilayers after AC (G) were observed in comparison to GI (H) and O_2_-NTP (I).

The adhesion and morphology of MG-63 cells seeded on sterilized disks of TA6V and PEEK were observed by SEM after 3 and 10 days of culture (Figs 4 and 5). At day 3, cells were mainly located in the center of the disks whatever the sterilization process (AC, GI and O_2_-NTP) (Figs 4A–4C and 5A–5C). No cell was observed near the border of the disks. After 10 days of culture, a cell population covering almost the entire surface of both materials was noted whatever the sterilization process (Figs 4D–4F and 5D–5F). This proliferation led to the formation of cellular multilayers which were less numerous after AC compared to GI and O_2_-NTP (Figs 4G–4I and 5G–5I). However, no changes in different cell morphology were noticed regardless of the sterilization methods. In all conditions, MG-63 were flattened cells tightly anchored to the substrate and to one another. A few secretory vesicles were observed at the cell surface.

## Discussion

AC and GI are the most common used methods to sterilize implants and consequently to prevent hospital-acquired infection and preserve human health. These methods aim to eliminate any microorganisms from medical devices and to preserve their sterile state at the end of the treatment. However, some medical devices cannot be subjected to those without altering their physical, topographical or biocompatibility properties. Consequently, NTP has been studied as an alternative standard sterilization process. Numerous studies have shown the efficacy of NTP sterilization using different processes such as dielectric barrier, plasma jet… [3, 4, 18, 21]. However, those processes cannot preserve the sterile state of items after the end of treatment. To overcome this issue, we have developed a new NTP sterilization process generating plasma inside a sealed bag containing the device [23–25]. The aim of this study was thus to investigate the effect of this new sterilization process on the cytocompatibility of two implantable materials. According to ISO 10993–5 norm [29], major hallmarks of cytocompatibility (i.e. proliferation, viability, toxicity and morphology) were assessed on MG-63 osteoblast-like cells seeded on these two materials used in dentistry or orthopedic fields.

The results of the present study suggest that there is no difference between the three sterilization processes. Whatever the treatment, the results of DNA quantification and WST-1 after 3, 7 and 10 days of culture suggested a similar increase in the MG-63 cells proliferation and viability on PEEK and TA6V. LDH showed low values on day 3 that slowly increased until day 10. This could be explained by LDH accumulation due to the medium not being renewed for 10 days and the high cell proliferation. As confirmed by SEM observations, this proliferation increased from day 3 to 10, leading to the coverage of almost the entire surface of both materials and to the formation of cellular multilayers. So, O_2_-NTP sterilization led to no cytotoxicity like AC and GI, but also promoted MG-63 cell viability and proliferation.

To our knowledge, only one study dealt with the cytocompatibility of biomaterials sterilized by different processes. Indeed, Park *et al*. [3] compared the effect of AC, GI, UV, and O_2_-NTP on titanium implant surface properties. The authors determined the response of MG-63 cells cultured on cleaned and re-sterilized TA6V disks. They studied three markers of cell differentiation: alkaline phosphatase as an early mineralization marker, osteocalcin as a later mineralization marker, and osteoprotegerin, which inhibits osteoclast differentiation. They highlighted that proliferation and differentiation of MG-63 cells were sensitive to all treatments. They concluded that the cellular responses were dependent on the surface properties of the titanium being modified by these treatments. Indeed, O_2_-NTP sterilization resulted in an increase of wettability and roughness of the titanium surface. GI sterilization resulted only in an increase in wettability. In contrast, AC led to a decrease in wettability and roughness of the titanium surface. This could explain our SEM observations showing a lesser surface coverage of TA6V sterilized by AC than GI and O_2_-NTP on culture day 10.

Few studies were carried out that only concentrated on understanding the cells response to biomaterials functionalized with different non-thermal plasma processes [2, 4, 10, 11, 30]. Two studies treated the effect of PEEK functionalization by NTP on the MG-63 cell adhesion and proliferation [2, 11]. They showed that an O_2_-NTP treatment increased the wettability and the roughness of PEEK surface improving the attachment of MG-63 cells. Likewise, Guo *et al*. [4] demonstrated the improvement of attachment, proliferation and viability of murine and human gingival fibroblasts on PEEK and also on titanium surfaces treated by O_2_-NTP. The same response of different cells such as gingival fibroblasts and osteoblasts was also observed on functionalized titanium according to Rabel *et al*. [10] and Henningsen *et al*. [9]. The latter correlated the osteoblast-like cells’ response only to the functionalization of the upper surface of titanium increasing the oxide layer thickness and the wettability. This present study was to assess only MG-63 osteoblast-like cells response, which could predict the hard tissues behavior surrounding implants, and not fibroblasts that could also provide some information about interactions with soft tissues [31–33]. Hence, to overcome this limitation, it will be interesting to evaluate the adhesion of primary human osteoblasts and fibroblasts on different implant materials sterilized by O_2_-NTP, which could lead to the modification of their surface chemistry and surface topography as well as their impact on cell behavior.

All these studies highlighted the improvement of viability and proliferation of various cells on titanium and PEEK materials sterilized or functionalized by O_2_-NTP. Moreover, the response of different cell types was similar, whatever the plasma process generated in different conditions such as atmospheric [11], low or medium pressure plasma generated into a vacuum chamber [2–4, 9, 10]. Even if the present study used an O_2_-NTP generated into a sealed bag under high vacuum conditions, the results were in accordance with the literature. Thus, the sealed bag and devices exposed to O_2_-NTP did not release cytotoxic particles for MG-63 osteoblast-like cells. Moreover, since O_2_-NTP led to similar cell responses as autoclave and gamma-irradiation, it could be a complementary process to the two usual sterilization methods.

## Conclusion

The three processes (AC, GI and O_2_-NTP) used to sterilize titanium and PEEK led to an optimized surface promoting the proliferation and viability of osteoblast-like cells. Therefore, this novel cost-less O_2_-NTP could help achieve an acceptable level of sterility assurance while functionalizing medical devices to improve their osseointegration. Further studies involving human primary osteoblast are necessary to determine physical and chemical modifications of titanium and PEEK surfaces after this sterilization and their impacts on these other cells.
